# Network Pharmacology-Based and Experimental Identification of the Effects of Quercetin on Alzheimer’s Disease

**DOI:** 10.3389/fnagi.2020.589588

**Published:** 2020-10-23

**Authors:** Pingfang Qi, Jing Li, Shichao Gao, Yirong Yuan, Yindi Sun, Na Liu, Yuanyuan Li, Gang Wang, Ling Chen, Jing Shi

**Affiliations:** ^1^Department of Pharmacy, The People’s Hospital of Yichun City, Yichun, China; ^2^Department of Pharmacy, The Affiliated Hospital of Qingdao University, Qingdao, China; ^3^Department of Clinical Laboratory, The People’s Hospital of Yichun City, Yichun, China; ^4^Department of Pharmaceutical Sciences, School of Pharmacy and Pharmaceutical Sciences, University at Buffalo, Buffalo, NY, United States; ^5^Department of Neurosurgery, The People’s Hospital of Yichun City, Yichun, China; ^6^Department of Traditional Medical Orthopedics, Honghui Hospital, Xi’an Jiaotong University, Xi’an, China; ^7^School of Pharmacy, Hangzhou Medical College, Hangzhou, China; ^8^Department of Clinical Pharmacology, Key Laboratory of Clinical Cancer Pharmacology and Toxicology Research of Zhejiang Province, Affiliated Hangzhou First People’s Hospital, Zhejiang University School of Medicine, Hangzhou, China

**Keywords:** bioinformatics analysis, experimental verification, AD biomarkers, AD diagnosis, treatment targets

## Abstract

Alzheimer’s disease (AD) is one of the neurodegenerative brain disorders inducing nearly half of dementia cases, and the diagnosis and treatment of AD are the primary issues clinically. However, there is a lack of effective biomarkers and drugs for AD diagnosis and therapeutics so far. In this study, bioinformatics analysis combined with an experimental verification strategy was used to identify the biomarkers and the quercetin targets for AD diagnosis and treatment. First, differentially expressed genes in the AD brain were identified by microarray data analysis. Second, quercetin, a predominant flavonoid, was used to screen the target genes. Third, the drug–disease network was determined, and the target genes of quercetin treatment were obtained in AD-related HT-22 cell-based assay. Six genes, including MAPT, PIK3R1, CASP8, DAPK1, MAPK1, and CYCS, were validated by the system pharmacology analysis in the hippocampus samples of AD patients. The results suggested that MAPT, PIK3R1, CASP8, and DAPK1 were significantly increased, but MAPK1 and CYCS were significantly decreased in HT-22 cells after Aβ1-42 treatment. Moreover, MAPK1 and CYCS were markedly increased, but MAPT, PIK3R1, CASP8, and DAPK1 were markedly decreased after quercetin treatment in these HT-22 cells. Altogether, MAPT, PIK3R1, CASP8, DAPK1, MAPK1, and CYCS are all the biomarkers for AD diagnosis and the targets of quercetin treatment, and our findings may provide valuable biomarkers for AD diagnosis and treatment.

## Introduction

Alzheimer’s disease (AD) is a neurodegenerative brain disease that is characterized by progressive cognitive impairment and memory loss, and psychiatric symptoms, which mostly induces over half of the dementia cases (Najafi et al., 2016). An estimated 115 million patients will be diagnosed with AD by 2050. These AD patients will progress to dementia within 5 years of diagnosis and will account for approximately half of all dementia cases (Luo et al., 2016). The typical pathogenesis of AD is the accumulation of amyloid-β (Aβ) aggregates, and the hyperphosphorylation of the tau proteins, which together lead to neurofibrillary tangles (NFTs) and synaptic dysfunction (Wallace and Dalton, 2011; Asaad and Lee, 2018). Current AD therapy is only for a single target, the effects are also limited, and always accompanied by many adverse effects (Aliev et al., 2008; Rountree et al., 2009). Therefore, an active compound against one or more of these triggering factors may be a promising strategy for the treatment of AD.

Phytochemical flavonoids are powerful eliminators of reactive oxygen and nitrogen species, which are associated with oxidative stress (Russo et al., 2012). Flavonoids have been proven beneficial in the prevention of neurodegenerative disorders and also may delay the progress of neurodegeneration (Solanki et al., 2015). Previous studies showed that the flavonoids are attributed to the reduction of Aβ toxicity and decreasing oxidative stress, which is important in ameliorating the pathogenesis of AD (Baptista et al., 2014; Deng et al., 2017).

Quercetin (3,5,7,30,40-penta hydroxyflavone), a predominant flavonoid, is one of the most effective antioxidants of plant origin and commonly in edible plants (Brüll et al., 2015). Quercetin has various beneficial effects on human health, such as anticarcinogenic, anti-inflammatory, and anti-infective effects. It also inhibits lipid peroxidation and platelet aggregation and stimulates mitochondrial biogenesis (Li et al., 2016). The quercetin can cross the blood–brain barrier (BBB) and exhibits antioxidant and anti-inflammatory properties in the brain (Youdim et al., 2004), which exerts the neuroprotective effects in neurodegenerative disorders, such as AD (Rishitha and Muthuraman, 2018) and Parkinson’s disease (PD) (El-Horany et al., 2016). Increasing evidence suggested that quercetin has shown therapeutic efficacy in multiple AD animal models, such as mouse and *Drosophila* models of AD (Richetti et al., 2011; Wang et al., 2014; Sabogal-Guáqueta et al., 2015). Despite a large number of studies on the biological activity of quercetin, the mechanisms of its action on the treatment of AD are still not well understood.

In this study, bioinformatics analyses, including microarray data analysis and an integrated system pharmacology approach, were used to examine the mechanisms of quercetin treatment. First, the quercetin was used to screen the potential target genes, and then, the differentially expressed genes of AD were identified using microarray datasets. Second, the quercetin target genes would be confirmed as the AD-related genes. Finally, the predicted target genes were verified by quantitative polymerase chain reaction (qPCR) with different doses of quercetin treatment in the HT-22 cells. Our study provides a more specific and effective way to offer new insight into the mechanisms of quercetin in the treatment of AD.

## Materials and Methods

### Quercetin Target Gene Identification

The chemical structure of quercetin was obtained from the PubChem database^1^. Afterward, the quercetin target genes were screened from the PharmMapper database^2^, the Similarity ensemble approach (SEA) database^3^, and the function of the target genes were analyzed by the Swiss Target Prediction database^4^.

### Microarray Data and Differentially Expressed Gene Analysis

Microarray dataset GSE5281 was downloaded from the Gene Expression Omnibus (GEO) database and collected using the GPL570 platform (Affymetrix Human Genome U133 Plus 2.0 Array). This microarray dataset formed the study of “Alzheimer’s disease and the normal aged brain.” This study aims to find the differentially expressed genes (DEGs) of the AD brain (Liang et al., 2008), which is consistent with the design of our study. Difference analysis was performed by R script using limma (Linear models for microarray analysis) R package, *p* < 0.05 and |logFC| > 1 as cutoff values for screening DEGs. The DEGs of the comparison group were shown as the volcano plot. The heatmap of the expression data was generated using ClustVis online tools^5^. The expression value of the DEGs was obtained from the GEO2R online tools.

### Network Establishment

The interaction of quercetin target genes and DEGs of AD patients were obtained by the Venn diagram. Cytoscape 3.7.2 was used to determine the drug–target–disease network. Furthermore, the STRING database^6^ was used to analyze the protein–protein interactions (PPIs) of the quercetin potential target genes, and the hub genes were counted by R script.

### GO and KEGG Enrichment Analysis

The GO and KEGG enrichment analysis was using the Database for Annotation, Visualization, and Integrated Discovery (DAVID) database^7^, which is an integrated online biological knowledge base and analytical tool. In this study, the target genes were mapped into DAVID and to identify the biological processes, cellular components, molecular function, and KEGG pathways of the predicted target genes involved. The map of the KEGG signaling pathway was obtained from the KEGG database^8^.

### Preparation of Treatment Reagents

The peptide Aβ1-42 (Invitrogen, Carlsbad, CA, United States) was completely dissolved in DMSO (Sigma, St. Louis, MO, United States), then cold Ham’s F12 was added (Caisson Labs, Smithfield, UT, United States) and incubated at 4°C overnight. The solution was then centrifuged at 14,000 × *g* × 10 min at 4°C. The supernatant was carefully transferred to a sterile tube. This Aβ1-42 stock solution was stored at -80°C and equilibrated for 1 h at room temperature before use. Quercetin (Acmec biochemical, Shanghai, China) was diluted to a final concentration of 100 μM in DMSO before use.

### Cell Lines and Treatment

The HT-22 cells were cultured in DMEM medium with 10% fetal bovine serum (FBS) and 1% penicillin–streptomycin (PS) and incubated at 37°C in 5% CO_2_ cell incubator. Cells were divided into four groups, normal control (NC), NC group treatment with quercetin, Aβ1-42 treatment, and Aβ1-42 group treatment with quercetin. When the cells were confluent (>90%), the fresh media containing various concentrations of drugs (10 μM Aβ1-42 for 12 h, and 100 μM quercetin for 48 h) were added to the carefully aspirated wells. Cells were then harvested and prepared for the following tests.

### Quantitative Polymerase Chain Reaction Analysis

The cells were collected and washed by 4°C PBS. Then, the RNeasy Mini kit (Qiagen, Germany) was used to extract the total RNA and reversed by ThermoScript RT-PCR System (Invitrogen, Carlsbad, CA, United States). The mRNA expression was detected by Talent qPCR kit (TIANGEN, Beijing, China). The primers used in this study are shown in Table 1.

**TABLE 1:** The primer sequences for real-time PCR.

Gene	Forward primer	Reverse primer
MAPT	5′-CGCTGGGCATGTGACTCAA-3′	5′-TTTCTTCTCGTCATTTCCTGTCC-3′
MAPK1	5′-GGTTGTTCCCAAATGCTGACT-3′	5′-CAACTTCAATCCTCTTGTGAGGG-3′
CASP8	5′-TGCTTGGACTACATCCCACAC-3′	5′-GTTGCAGTCTAGGAAGTTGACC-3′
PIK3R1	5′-ACACCACGGTTTGGACTATGG-3′	5′-GGCTACAGTAGTGGGCTTGG-3′
CYCS	5′-CCAAATCTCCACGGTCTGTTC-3′	5′-ATCAGGGTATCCTCTCCCCAG-3′
DAPK1	5′-ATGACTGTGTTCAGGCAGGAA-3′	5′-CCGGTACTTTTCTCACGACATTT-3′
β-actin	5′-GGCTGTATTCCCCTCCATCG-3′	5′-CCAGTTGGTAACAATGCCATGT-3′

### Statistical Analysis

Statistical analysis was calculated by GraphPad Prism software version 7.0 (CA, United States). All data were displayed as the mean ± SEM. The comparison of the two groups was analyzed using the two-tailed Student’s *t*-test. A value of *p* < 0.05 was considered significant.

## Results

### Prediction of the Quercetin Target Genes

**FIGURE 1:**
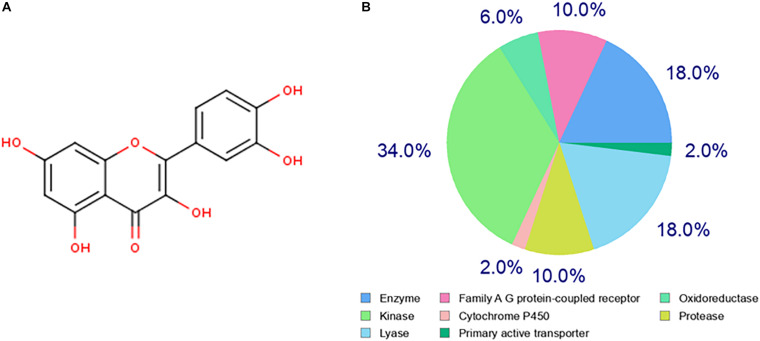
Prediction of the quercetin target genes. **(A)** The chemical structure of the quercetin. **(B)** The classification of drug target genes according to their biochemical criteria. Different colors indicate different items.

The chemical structure of quercetin was obtained from the PubChem database as shown in Figure 1A. Based on its structure, PharmMapper database, SEA database, and Swiss Target Prediction database were used to predict the potential target genes of quercetin. A total of 277 potential target genes were obtained from those databases, and then, the function of the target genes was classified according to their biochemical criteria, as shown in Figure 1B. These 277 targets mainly include kinase, enzyme, lyase, protease, family A/G protein-coupled receptors, etc.

### Identification of Differentially Expressed Genes of AD Patients

**FIGURE 2:**
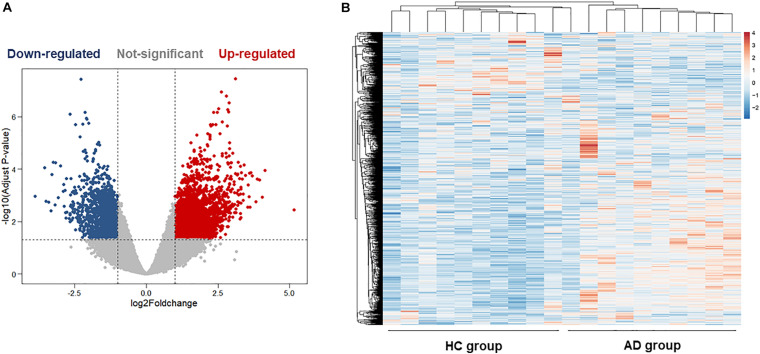
Bioinformatics analysis of differentially expressed genes (DEGs) in the hippocampus of health control (HC) and Alzheimer’s disease (AD) patients. **(A)** The volcano plot of DEGs in the hippocampus between the HC group and the AD group. **(B)** Heatmaps of DEGs in the hippocampus of the HC group and the AD group. Colors in the heatmaps indicate the row Z-score among the different datasets. High expression is shown by the red color, and low expression is shown by the blue color.

The hippocampus gene expression datasets GSE5821 was downloaded from the GEO database for analyzing the AD-related gene changes. The gene expression analysis was made on health control and AD groups. As shown in Figures 2A,B, there were a total of 3,256 differentially expressed genes with 1,227 upregulated and 2029 downregulated in AD patients, which may be closely related to the progression of AD.

### Prediction of Alzheimer’s Disease-Related Quercetin Target Genes by System Pharmacology Approach

**FIGURE 3:**
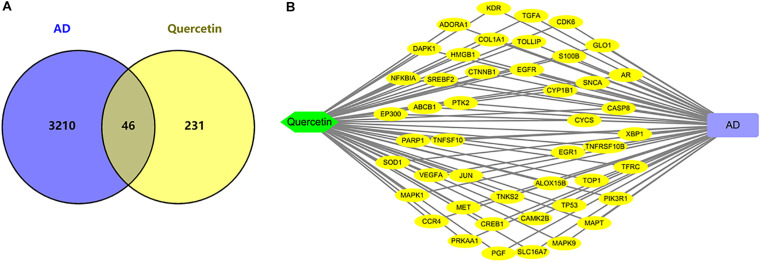
Network analysis of quercetin target genes and AD differentially expressed genes. **(A)** Venn diagram of quercetin target genes and AD differentially expressed genes. **(B)** The network of Quercetin, AD, and all the potential target genes. Yellow nodes represented the target genes.

As shown in Figure 3, we found 277 potential target genes of quercetin treatment, and 3,256 differentially expressed genes of AD. To further analyze the quercetin’s effects on AD, the interaction of drug target genes and AD-related genes was used as a Venn diagram. As shown in Figure 3A, there were 46 genes in both groups, and these target genes were not only AD-related genes but also the drug targets. At the same time, an interactive Quercetin-target gene–AD network was constructed (Figure 3B), which indicated that quercetin might have effect on AD by stimulating or inhibiting these target genes.

### The Protein–Protein Interaction Analysis of the Quercetin Target Genes

**FIGURE 4:**
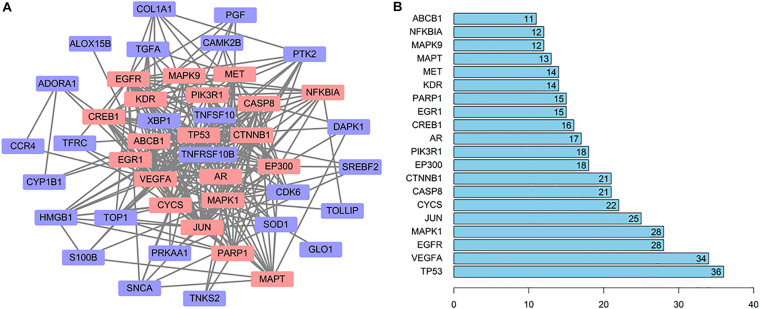
The interaction network of the quercetin target genes. **(A)** The protein—protein interaction (PPI) network of the quercetin target genes. The orange nodes are the hub genes in the network. **(B)** Bar plot of the number of hub gene links.

In order to explore the relationship between these 46 potential target genes, the protein–protein interaction (PPI) analysis was done by the STRING database. A network was generated, the hub genes were analyzed, the top 20 hub genes in the network were marked in red, and other genes connected with hub genes were presented by purple nodes (Figure 4A). A bar plot of the number of hub gene links is shown in Figure 4B, and we found that the hub genes, such as TP53, MAPK1, CYCS, CASP8, PIK3R1, and MAPT, may play a critical role in the biological activity in the quercetin treatment process.

### Functional Enrichment Analysis for the Quercetin Target Genes

**FIGURE 5:**
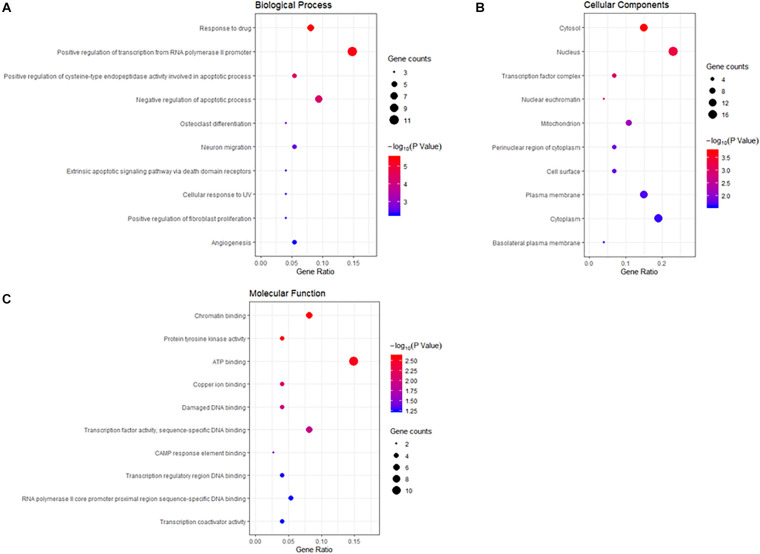
Gene ontology (GO) enrichment analysis of the quercetin target genes. **(A)** The plot of enriched biological processes. **(B)** The plot of enriched cellular components. **(C)** The plot of enriched molecular functions. The number of genes enriched in each GO term is shown as the circle size, the *p*-value shown as different colors.

**FIGURE 6:**
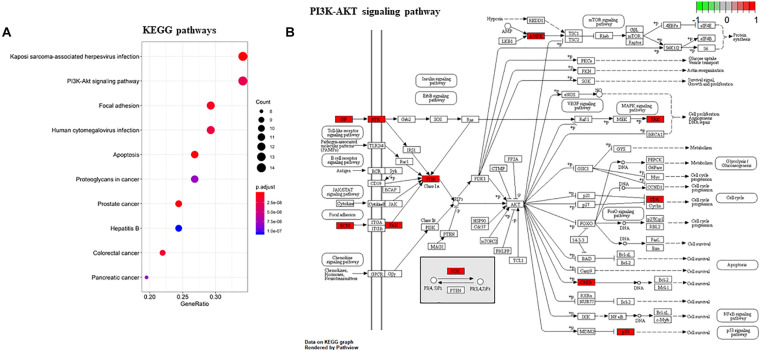
KEGG enrichment analysis of the quercetin target genes. **(A)** KEGG annotation of target genes. The number of genes enriched in each KEGG term is shown as the circle size, the *p*-value shown as different colors. **(B)** The important genes were mainly distributed in the PI3K–AKT signaling pathway. Arrows represent activation effect, T-arrows represent inhibition effect and segments show activation effect or inhibition effect. The red nodes are the intersection genes.

To gain a comprehensive understanding of these target genes, the DAVID database was used to do the GO and KEGG enrichment analysis. A variety of GO enrichment terms were enriched, including 76 biological processes, 13 cellular components, and 12 molecular functions. The top 10 GO terms are shown in Figure 5. We found that the biological processes such as negative regulation of apoptotic process and neuronal migration (Figure 5A), cellular components like nucleus and mitochondrion (Figure 5B), and molecular function like chromatin binding and ATP binding (Figure 5C), were enriched, which may be involved in the biological activity in the quercetin treatment process. In addition, 75 KEGG pathways were enriched (Figure 6A), and the important genes were mainly distributed in the PI3K–AKT signaling pathway (Figure 6B), which indicate that quercetin might ameliorate AD-related symptoms through the PI3K–AKT signaling pathway.

### The Expression Changes of Selected Quercetin Target Genes in the Alzheimer’s Disease Process

**FIGURE 7:**
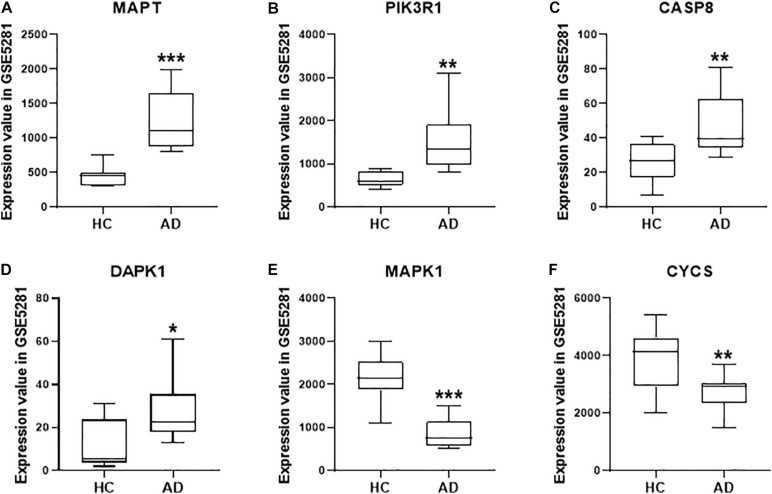
The quercetin target gene expression in HC and AD groups of GSE5281 dataset. The expression of MAPT **(A)**, PIK3R1 **(B)**, CASP8 **(C)**, DAPK1 **(D)**, MAPK1 **(E)**, and CYCS **(F)** in different groups. Values are presented mean ± SEM (*n* = 10 in each group). ^∗^*p* < 0.05, ^∗∗^*p* < 0.01, ^∗∗∗^*p* < 0.001 vs. HC group.

Based on the system pharmacology analysis of quercetin target genes, and the results of the network and functional enrichment analysis, six genes, including MAPT, PIK3R1, CASP8, DAPK1, MAPK1, and CYCS, were selected to validate the AD treatment effects of the quercetin. As shown in Figure 7, MAPT, PIK3R1, CASP8, and DAPK1 were significantly increased (*p* < 0.05, Figures 7A–D), but MAPK1 and CYCS were significantly decreased (*p* < 0.05, Figures 7E,F) in the AD group of the GSE5281 dataset.

**FIGURE 8:**
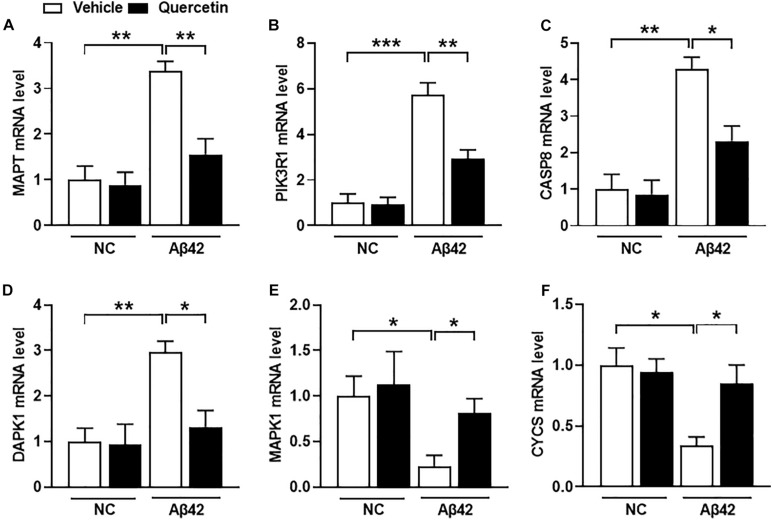
The quercetin potential target genes among different groups of HT22 cells by quantitative polymerase chain reaction (qPCR) analysis. The mRNA level of MAPT **(A)**, PIK3R1 **(B)**, CASP8 **(C)**, DAPK1 **(D)**, MAPK1 **(E)**, and CYCS **(F)** in different treatment groups. Data were normalized to β-actin, and values are presented as mean ± SEM (*n* = 5 in each group). ^∗^*p* < 0.05, ^∗∗^*p* < 0.01, ^∗∗∗^*p* < 0.001.

To further validate whether these six genes are related to AD pathology, we performed a serial of qPCR quantification in the HT-22 cells in the presence of Aβ1-42. As shown in Figure 8, the mRNA levels of MAPT, PIK3R1, CASP8, and DAPK1 were significantly increased (Figures 8A–D, *p* < 0.05), and MAPK1 and CYCS were significantly decreased (Figures 8E,F, *p* < 0.05), after treatment of the HT22 cells with Aβ1-42, which were consistent with the GSE5281 dataset results. Moreover, the mRNA levels of MAPT, PIK3R1, CASP8, and DAPK1 were significantly decreased (Figures 8A–D, *p* < 0.05), and MAPK1 and CYCS were significantly increased (Figures 8E,F, *p* < 0.05), after treatment of these HT22 cells with quercetin for 48 h. These results support that all six genes are closely related to the AD process as the quercetin treatment targets.

## Discussion

Alzheimer’s disease is closely related to neuroinflammation, impairment of cerebral circulation, altered synaptic function, and cerebral amyloid angiopathy, and these typical pathological changes are considered to be important drug targets for AD treatment (Ghanemi, 2015). There is a lot of evidence that suggests that some dietary supplements could minimize the risk of AD (Hartman et al., 2006). The flavonol quercetin, which can be found in various fruits and vegetables, has received extensive attention due to its potential biological activities (Cooper and Ma, 2017). Some studies suggested that quercetin can protect neurons against the oxidative stress by regulating the process of apoptosis in the neuronal cells (Ansari et al., 2009). Other studies found that quercetin improves cholinergic function and acts as a neuroprotective agent in neurodegenerative diseases (Gupta et al., 2016). Recent studies have been suggested that quercetin may inhibit the NFT formation by attenuating the Aβ aggregation (Zhang et al., 2016). However, the molecular mechanisms of quercetin to ameliorate AD are still unknown.

In our study, we examined the AD-related quercetin target genes through a series of bioinformatics analysis combined with the subsequent experimental verification by real-time RT-PCR assay. Based on these strategies, we obtained 46 genes, which were related to AD pathology and quercetin treatment. To further understand the function of these genes, the PPI analysis and functional enrichment analysis was conducted afterward. We found that the hub genes, such as TP53, MAPK1, CYCS, CASP8, PIK3R1, and MAPT, may play a critical role in the biological pathway in the quercetin treatment process. The negative regulation of the apoptotic process, ATP binding, mitochondrion, and PI3K–AKT signaling pathway may also be involved in the biological activity in the quercetin treatment process. After analysis of these 46 genes by network and functional enrichment analysis, six genes, including MAPT, PIK3R1, CASP8, DAPK1, MAPK1, and CYCS, were chosen for experimental validation. We found that all the six genes were verified to be differentially expressed in the GSE5281 dataset and after the Aβ1-42 treatment. Meanwhile, all the six genes were markedly changed after quercetin treatment, suggesting that they might serve as potential targets in the progression of AD and quercetin treatment.

The microtubule-associated protein tau (MAPT) is an intracellular tau protein mainly restricted to axons in mature neurons (Binder et al., 1985; Kosik et al., 1986), which plays an important role in assembling and stabilizing of microtubules (Mandelkow and Mandelkow, 2012). The aberrant aggregation of MAPT in neurons induced many irreversible, progressive neurodegeneration diseases, such as AD, corticobasal degeneration (CBD), and frontotemporal dementia with parkinsonism linked to chromosome 17 (FTDP-17) (Guo et al., 2017). The MAPT, mislocated to the somatodendritic compartments, is one of the early signs of AD neurodegeneration (Braak et al., 1994; Balaji et al., 2018). In addition, the accumulation of MAPT in dendrites is a critical step in Aβ-induced neurotoxicity (Ittner et al., 2010; Zempel et al., 2010). In this study, we predicted and verified that the level of MAPT was significantly increased in the brain sample of AD patients and after Aβ1-42 treatment in HT-22 cells. Moreover, quercetin treatment can inhibit MAPT expression, which indicates that MAPT is the target in the treatment of quercetin in AD patients. Therefore, it is important to explore the pathological changes of MAPT in the progression of AD, which also has an important implication for tau-targeting therapeutics (Strang et al., 2019).

Death-associated protein kinase 1 (DAPK1) is a death domain-containing and stress-responsive serine/threonine protein kinase (Bialik and Kimchi, 2006). Via its death domain, DAPK1 is a positive mediator of apoptosis-like cell death and can be induced by a variety of death receptor activations (Stevens and Hupp, 2008). The previous study showed that DAPK1 deletion mice can enhance the learning and spatial memory (Yukawa et al., 2006), which may be through the regulation of DAPK1 in tau toxicity by modulating microtubule (MT) assembly and neuronal differentiation (Wu et al., 2011). However, the association between DAPK1 and AD has not been sufficiently explored, and DAPK1 regulation in AD progression is poorly understood. In the present study, our results demonstrated that DAPK1 expression is increased in AD patients and after the Aβ1-42 treatment in HT-22 cells, which can be inhibited by quercetin treatment. This suggests that DAPK1 may be a novel therapeutic target for treating human AD and other tau-related pathologies as a valuable biomarker of the progression of AD.

Caspase-8 (CASP8) belongs to the cysteine–aspartic acid protease (caspase) family and plays a pivotal initiator role in the death receptor pathway of apoptosis (Ashkenazi and Dixit, 1999). CASP8 is thought to activate downstream caspases, particularly caspase-3, which is an early event in the apoptotic cascade (Takahashi, 1999). Caspase-3 is involved in the APP proteolysis (Gervais et al., 1999) and increased in the AD brains (Su et al., 2001), which indicates that CASP8 might be activated in the AD brain (Stadelmann et al., 1999). CASP8 was reported to mediate Aβ-induced neuronal apoptosis in an animal study (Ivins et al., 1999), and Aβ may activate CASP8 through the cross-linking and activation of receptors of the death receptor family, thereby inducing neuronal apoptosis of AD brain (Rohn et al., 2001). In the present study, CASP8 was significantly increased in the AD brain and Aβ1-42-treated HT-22 cells, which can be inhibited after quercetin treatment. This indicates that CASP8 has the function to promote the AD process as the target for quercetin treatment. Although the function of caspases in AD has been proposed, the role of CASP8 in AD still requires further studies.

Mitogen-activated protein kinase 1 (MAPK1), a serine/threonine kinase, is one of the components of the MAPK signaling pathway, which can activate many cellular effects, including the regulation of cell growth, survival, and differentiation (Fang and Richardson, 2005). MAPK1 can induce the overactivation of Dynamin-related protein 1 (Drp1), the mitochondrial fission protein, leading to mitochondrial fragmentation, neuronal apoptosis (Pyakurel et al., 2015), and neurodegeneration (Roe and Qi, 2018). The cytochrome c, somatic (CYCS) is the isoform of cytochrome c (Cyt c), as an integral membrane protein located in the mitochondrial intermembrane, and participant in ATP synthesis of the mitochondria (Ow et al., 2008), and also play an important role in apoptosis (Zhivotovsky et al., 1998). Based on the function in mitochondria energy metabolism as well as in apoptosis, MAPK1 and CYCS were chosen as the candidate genes to investigate the AD progress and quercetin treatment. In the present study, MAPK1 and CYCS were significantly decreased in AD patients and the Aβ1-42-treated HT-22 cells, which can be reversed by quercetin treatment. This indicates that MAPK1 and CYCS were involved in the progression of AD and the quercetin treatment.

Phosphoinositide-3-kinase, regulatory subunit 1 (PIK3R1) is one of the isoforms of phosphoinositide 3-kinase (PI3K). PI3K is a heterodimer that consists of the subunit of p85 and p110 (Ueki et al., 2000). The PIK3R1 gene encodes the PI3K catalytic subunit p85α in most eukaryotic cells (Taniguchi et al., 2006). PI3K pathway plays an essential role in proliferation, apoptosis, and metabolism of both normal and malignant cells (Vanhaesebroeck et al., 2012). It has been reported that PIK3R1 is differentially expressed in many human cancers and is closely related to tumor progression and metastasis (Lin et al., 2015), which make it an important therapeutic target through inhibiting the PI3K/AKT/mTOR pathway (Lahusen et al., 2009). However, the role of PIK3R1 in the AD progress and quercetin treatment remains blank. In this study, we first demonstrated that PIK3R1 was significantly increased after Aβ1-42 treatment, and it was also the target of quercetin treatment. Further studies that focus on the roles of PIK3R1 in the diagnosis and treatment of AD patients are essential and of great interest.

This bioinformatics analysis combined with the experimental verification strategy provides six potential quercetin target genes for the AD treatment, which is the first study to use such an approach for predicting quercetin treatment targets. The limitation of our study is that only HT-22 cells treated with Aβ1-42 monomer were used to simulate the AD status. Alternative approaches, using human neuronal cells, such as iPS-derived neurons, and Aβ1-42 oligomer/aggregate rather than monomer, as well as AD animal models, would further support our findings in the future.

## Conclusion

A total of 277 target genes of quercetin and 3,256 differentially expressed genes in AD were obtained in our study, and then the functional analysis suggested that six genes (MAPT, PIK3R1, CASP8, DAPK1, MAPK1, and CYCS) were involved in the progression of AD and the treatment target of AD patients. Our study provides valuable information to investigate the pathogenesis of AD and the potential mechanisms of quercetin in the treatment of AD.

## Data Availability Statement

The datasets presented in this study can be found in online repositories. The names of the repository/repositories and accession number(s) can be found in the article/supplementary material.

## Author Contributions

PQ and JL wrote and prepared the original draft. SG and YY were responsible for the software and data curation. YS and NL were in charge of the resources and validation. YL and GW wrote, reviewed and edited the manuscript. LC and JS were responsible for the conceptualization, funding acquisition, and supervision. All authors approved the manuscript for publication.

## Conflict of Interest

The authors declare that the research was conducted in the absence of any commercial or financial relationships that could be construed as a potential conflict of interest.

## Footnotes

1 https://pubchem.ncbi.nlm.nih.gov/

2 http://www.lilab-ecust.cn/pharmmapper/

3 http://sea.bkslab.org/

4 http://www.swisstargetprediction.ch/

5 https://biit.cs.ut.ee/clustvis/

6 https://string-db.org/

7 https://david.ncifcrf.gov/

8 https://www.genome.jp/kegg/pathway.html
